# *N*-[(2,4-Dimethyl­phen­yl)carbamothio­yl]-2-methyl­benzamide

**DOI:** 10.1107/S1600536808009501

**Published:** 2008-04-10

**Authors:** B. M. Yamin, S. Yousuf, M. S. M. Yusof, R. H. Jusoh

**Affiliations:** a School of Chemical Sciences and Food Technology, Universiti Kebangsaan Malaysia, UKM 43650 Bangi Selangor, Malaysia; b HEJ Research Institute of Chemistry, International Center for Chemical and Biological Sciences, University of Karachi, Karachi 75270, Pakistan; c Department of Chemistry, Universiti Malaysia Terengganu, Manngabang Telipot, Terengganu, Malaysia

## Abstract

The title compound, C_17_H_18_N_2_OS, adopts a *trans*–*cis* geometry of the thio­urea group which is stabilized by intra­molecular hydrogen bonds between the O atom of the carbonyl group and the H atom of the thio­amide group. A C—H⋯S intramolecular hydrogen bond is also present. In the crystal structure, mol­ecules are linked by inter­molecular N—H⋯S hydrogen bonds to form centrosymmetric dimers.

## Related literature

For the crystal structure of 1-(2,3-dimethyl­phen­yl)-3-(2-methyl­benzo­yl)thio­urea, which is isomeric with the title compound, see: Khawar Rauf *et al.* (2007 ▶). For bond-length data, see: Allen *et al.* (1987 ▶).
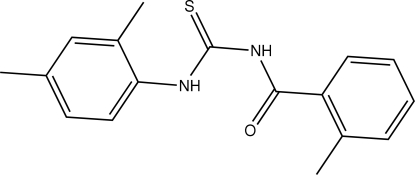


## Experimental

### 

#### Crystal data

C_17_H_18_N_2_OS
*M* *_r_* = 298.39
Triclinic, 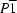

*a* = 6.2569 (15) Å
*b* = 9.862 (2) Å
*c* = 13.986 (3) Å
α = 69.461 (4)°
β = 86.199 (4)°
γ = 75.206 (4)°
*V* = 781.1 (3) Å^3^
*Z* = 2
Mo *K*α radiation
μ = 0.21 mm^−1^
*T* = 298 (2) K
0.27 × 0.18 × 0.09 mm

#### Data collection

Bruker SMART APEX CCD area-detector diffractometer
Absorption correction: multi-scan (*SADABS*; Bruker, 2000 ▶) *T*_min_ = 0.946, *T*_max_ = 0.982
7817 measured reflections
2904 independent reflections
2069 reflections with *I* > 2σ(*I*)
*R*_int_ = 0.034

#### Refinement

*R*[*F* ^2^ > 2σ(*F* ^2^)] = 0.047
*wR*(*F* ^2^) = 0.119
*S* = 1.02
2904 reflections
193 parameters
H-atom parameters constrained
Δρ_max_ = 0.23 e Å^−3^
Δρ_min_ = −0.17 e Å^−3^

### 

Data collection: *SMART* (Bruker, 2000 ▶); cell refinement: *SAINT* (Bruker, 2000 ▶); data reduction: *SAINT*; program(s) used to solve structure: *SHELXS97* (Sheldrick, 2008 ▶); program(s) used to refine structure: *SHELXL97* (Sheldrick, 2008 ▶); molecular graphics: *SHELXTL* (Sheldrick, 2008 ▶); software used to prepare material for publication: *SHELXTL*, *PARST* (Nardelli, 1995 ▶) and *PLATON* (Spek, 2003 ▶).

## Supplementary Material

Crystal structure: contains datablocks global, I. DOI: 10.1107/S1600536808009501/at2557sup1.cif

Structure factors: contains datablocks I. DOI: 10.1107/S1600536808009501/at2557Isup2.hkl

Additional supplementary materials:  crystallographic information; 3D view; checkCIF report

## Figures and Tables

**Table 1:** Hydrogen-bond geometry (Å, °)

*D*—H⋯*A*	*D*—H	H⋯*A*	*D*⋯*A*	*D*—H⋯*A*
N2—H2⋯O1	0.86	2.03	2.706	135
C17—H17*B*⋯S1	0.96	2.80	3.496	130
N1—H1⋯S1^i^	0.86	2.57	3.372	155

Symmetry code: (i) 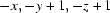.

**Fig. 1.**
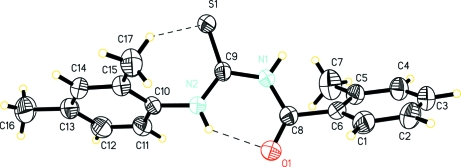
The molecular sStructure of (I) with displacement ellipsoids drawn at 50% probability level. The dashed lines indicates the intramolecular hydrogen bonds.

**Fig. 2.**
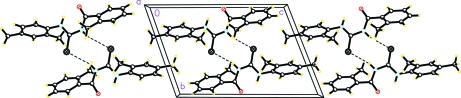
A packing diagram of (I). Hydrogen bonds are shown by dashed lines.

## supplementary crystallographic information

### Comment

The title compound, (I), is isomeric to the previously reported 1-(2,3-dimethylphenyl)-3-(2-methylbenzoyl)thiourea (II), (Khawar Rauf *et al.*, 2007) with the difference that the 2,3-dimethylphenyl ring is replaced by 2,4-dimethylphenyl (Fig.1). The bond lengths and angles are in normal range (Allen *et al.*, 1987) and in agreement with those in (II). The central thiourea moiety (S1/N1/N2/C9), 2-methylbenzoyl (C1—C8), and 2,3-dimethylphenyl (C10—C15) rings are each planar with a maximum deviation of 0.040 (2)Å for C8 atom from the least square plane. The dihedral angles between the thiourea moiety and the 2-methylbenzoyl and 2,3-dimethylphenyl rings are 52.96 (11) and 70.34 (12)°, respectively. The *trans*-*cis* geometry of the thiourea moiety is stabilized by N2—H2···O1 and C17—H17B···S1 intramolecular hydrogen bonds. In the crystal structure, the molecules are linked to form dimers by the N1—H1···S1 intermolecular hydrogen bond (symmtery codes as in Table 2) and arranged parallel to *c* axis (Fig.2).

### Experimental

The mixture of 2-methylbenzoyl chloride (9.720 g, 0.025 mol) with the equimolar amount of ammonium thiocyanate (1.903 g, 0.025 mol) and 2,3-dimethyl aniline (3.025 g, 0.025 mol) in 40 ml dry acetone was refluxed with stirring for 4 h. The solution was filtered and left to evaporate at room temperature. The colourless crystals obtained after a few days, was found suitable for X-ray investigations. The yield was 85% with melting point 413.2–415.7 K.

### Refinement

H atoms on the C and N parent atoms were positioned geomatrically, with C—H = 0.96, 0.93 and N—H = 0.86Å

and constrained to ride on their parent atoms with *U*_iso_(H) = 1.2*U*_eq_(CH and NH) and 1.5*U*_eq_(CH_3_).

### Crystal data

**Table:**

C_17_H_18_N_2_OS	*Z* = 2
*M**_r_* = 298.39	*F*_000_ = 316
Triclinic, *P*1	*D*_x_ = 1.269 Mg m^−^^3^
Hall symbol: -P 1	Mo *K*α radiation λ = 0.71073 Å
*a* = 6.2569 (15) Å	Cell parameters from 1377 reflections
*b* = 9.862 (2) Å	θ = 1.5–25.5º
*c* = 13.986 (3) Å	µ = 0.21 mm^−^^1^
α = 69.461 (4)º	*T* = 298 (2) K
β = 86.199 (4)º	Slab, colourless
γ = 75.206 (4)º	0.27 × 0.18 × 0.09 mm
*V* = 781.1 (3) Å^3^	

### Data collection

**Table:**

Bruker SMART APEX CCD area-detector diffractometer	2904 independent reflections
Radiation source: fine-focus sealed tube	2069 reflections with *I* > 2σ(*I*)
Monochromator: graphite	*R*_int_ = 0.034
Detector resolution: 83.66 pixels mm^-1^	θ_max_ = 25.5º
*T* = 298(2) K	θ_min_ = 1.5º
ω scans	*h* = −7→7
Absorption correction: multi-scan(SADABS; Bruker, 2000)	*k* = −11→11
*T*_min_ = 0.946, *T*_max_ = 0.982	*l* = −16→16
7817 measured reflections	

### Refinement

**Table:**

Refinement on *F*^2^	Secondary atom site location: difference Fourier map
Least-squares matrix: full	Hydrogen site location: inferred from neighbouring sites
*R*[*F*^2^ > 2σ(*F*^2^)] = 0.047	H-atom parameters constrained
*wR*(*F*^2^) = 0.119	*w* = 1/[σ^2^(*F*_o_^2^) + (0.0567*P*)^2^ + 0.1084*P*] where *P* = (*F*_o_^2^ + 2*F*_c_^2^)/3
*S* = 1.02	(Δ/σ)_max_ = 0.001
2904 reflections	Δρ_max_ = 0.23 e Å^−^^3^
193 parameters	Δρ_min_ = −0.17 e Å^−^^3^
Primary atom site location: structure-invariant direct methods	Extinction correction: none

### Special details

**Table:**

Geometry. All e.s.d.'s (except the e.s.d. in the dihedral angle between two l.s. planes) are estimated using the full covariance matrix. The cell e.s.d.'s are taken into account individually in the estimation of e.s.d.'s in distances, angles and torsion angles; correlations between e.s.d.'s in cell parameters are only used when they are defined by crystal symmetry. An approximate (isotropic) treatment of cell e.s.d.'s is used for estimating e.s.d.'s involving l.s. planes.
Refinement. Refinement of *F*^2^ against ALL reflections. The weighted *R*-factor *wR* and goodness of fit *S* are based on *F*^2^, conventional *R*-factors *R* are based on *F*, with *F* set to zero for negative *F*^2^. The threshold expression of *F*^2^ > σ(*F*^2^) is used only for calculating *R*-factors(gt) *etc*. and is not relevant to the choice of reflections for refinement. *R*-factors based on *F*^2^ are statistically about twice as large as those based on *F*, and *R*- factors based on ALL data will be even larger.

### Fractional atomic coordinates and isotropic or equivalent isotropic displacement parameters (Å^2^)

**Table:**

	*x*	*y*	*z*	*U*_iso_*/*U*_eq_	
S1	0.00124 (10)	0.51524 (6)	0.34145 (4)	0.0441 (2)	
O1	0.2196 (3)	0.02671 (18)	0.54848 (13)	0.0630 (5)	
N1	0.1412 (3)	0.27939 (19)	0.50519 (13)	0.0374 (4)	
H1	0.1341	0.3473	0.5313	0.045*	
N2	0.0595 (3)	0.22917 (19)	0.36460 (13)	0.0394 (5)	
H2	0.0883	0.1375	0.4048	0.047*	
C1	0.2131 (4)	0.0521 (3)	0.75752 (18)	0.0519 (6)	
H1A	0.1042	0.0051	0.7537	0.062*	
C2	0.2787 (5)	0.0473 (3)	0.85127 (19)	0.0644 (8)	
H2A	0.2120	−0.0007	0.9105	0.077*	
C3	0.4424 (5)	0.1138 (3)	0.8562 (2)	0.0639 (8)	
H3	0.4864	0.1112	0.9192	0.077*	
C4	0.5414 (4)	0.1835 (3)	0.77013 (19)	0.0541 (7)	
H4	0.6543	0.2264	0.7754	0.065*	
C5	0.4783 (4)	0.1923 (3)	0.67457 (17)	0.0430 (6)	
C6	0.3089 (3)	0.1265 (2)	0.66943 (16)	0.0367 (5)	
C7	0.5968 (5)	0.2679 (4)	0.5822 (2)	0.0696 (8)	
H7A	0.5020	0.3625	0.5433	0.104*	
H7B	0.6355	0.2060	0.5407	0.104*	
H7C	0.7286	0.2835	0.6034	0.104*	
C8	0.2225 (4)	0.1361 (2)	0.56942 (17)	0.0393 (5)	
C9	0.0679 (3)	0.3321 (2)	0.40383 (15)	0.0334 (5)	
C10	0.0056 (4)	0.2603 (2)	0.25979 (16)	0.0366 (5)	
C11	0.1738 (4)	0.2193 (3)	0.19879 (18)	0.0473 (6)	
H11	0.3167	0.1740	0.2259	0.057*	
C12	0.1297 (4)	0.2456 (3)	0.09727 (19)	0.0538 (7)	
H12	0.2440	0.2181	0.0565	0.065*	
C13	−0.0810 (4)	0.3119 (3)	0.05573 (17)	0.0499 (6)	
C14	−0.2460 (4)	0.3481 (3)	0.11976 (17)	0.0463 (6)	
H14	−0.3895	0.3910	0.0929	0.056*	
C15	−0.2098 (4)	0.3239 (2)	0.22153 (16)	0.0394 (5)	
C16	−0.1300 (5)	0.3419 (3)	−0.05520 (19)	0.0748 (9)	
H16A	−0.0953	0.4338	−0.0968	0.112*	
H16B	−0.2839	0.3494	−0.0643	0.112*	
H16C	−0.0420	0.2614	−0.0750	0.112*	
C17	−0.3997 (4)	0.3598 (3)	0.28774 (19)	0.0564 (7)	
H17A	−0.5362	0.3917	0.2493	0.085*	
H17B	−0.3828	0.4383	0.3099	0.085*	
H17C	−0.4015	0.2723	0.3462	0.085*	

### Atomic displacement parameters (Å^2^)

**Table:**

	*U*^11^	*U*^22^	*U*^33^	*U*^12^	*U*^13^	*U*^23^
S1	0.0630 (4)	0.0333 (3)	0.0343 (3)	−0.0086 (3)	−0.0061 (3)	−0.0108 (2)
O1	0.1010 (14)	0.0356 (9)	0.0529 (11)	−0.0138 (9)	−0.0262 (10)	−0.0130 (8)
N1	0.0502 (11)	0.0303 (9)	0.0333 (10)	−0.0070 (8)	−0.0101 (8)	−0.0128 (8)
N2	0.0524 (12)	0.0300 (9)	0.0347 (10)	−0.0063 (8)	−0.0104 (8)	−0.0107 (8)
C1	0.0657 (16)	0.0445 (14)	0.0448 (15)	−0.0180 (12)	−0.0029 (12)	−0.0108 (12)
C2	0.092 (2)	0.0591 (17)	0.0327 (14)	−0.0153 (16)	0.0040 (14)	−0.0072 (13)
C3	0.089 (2)	0.0587 (17)	0.0398 (16)	−0.0019 (16)	−0.0202 (15)	−0.0193 (13)
C4	0.0576 (16)	0.0573 (16)	0.0483 (16)	−0.0066 (13)	−0.0184 (13)	−0.0214 (13)
C5	0.0421 (13)	0.0453 (14)	0.0402 (14)	−0.0053 (11)	−0.0066 (11)	−0.0157 (11)
C6	0.0424 (13)	0.0311 (11)	0.0339 (12)	−0.0037 (10)	−0.0052 (10)	−0.0109 (10)
C7	0.0598 (17)	0.101 (2)	0.0598 (18)	−0.0409 (16)	0.0074 (14)	−0.0278 (17)
C8	0.0450 (13)	0.0363 (12)	0.0380 (13)	−0.0111 (10)	−0.0058 (10)	−0.0127 (10)
C9	0.0319 (11)	0.0387 (12)	0.0313 (12)	−0.0077 (9)	−0.0019 (9)	−0.0144 (10)
C10	0.0494 (14)	0.0326 (11)	0.0315 (12)	−0.0110 (10)	−0.0056 (10)	−0.0135 (9)
C11	0.0478 (14)	0.0472 (14)	0.0487 (15)	−0.0026 (11)	−0.0052 (12)	−0.0239 (12)
C12	0.0579 (17)	0.0569 (16)	0.0475 (15)	−0.0036 (13)	0.0052 (13)	−0.0274 (13)
C13	0.0694 (17)	0.0452 (14)	0.0338 (13)	−0.0083 (12)	−0.0056 (12)	−0.0152 (11)
C14	0.0504 (14)	0.0455 (14)	0.0404 (14)	−0.0049 (11)	−0.0128 (11)	−0.0143 (11)
C15	0.0428 (13)	0.0400 (12)	0.0361 (13)	−0.0090 (10)	−0.0031 (10)	−0.0141 (10)
C16	0.101 (2)	0.080 (2)	0.0370 (15)	−0.0053 (17)	−0.0084 (15)	−0.0226 (14)
C17	0.0470 (15)	0.0767 (19)	0.0493 (16)	−0.0126 (13)	−0.0008 (12)	−0.0279 (14)

### Geometric parameters (Å, °)

**Table:**

S1—C9	1.660 (2)	C7—H7A	0.9600
O1—C8	1.218 (3)	C7—H7B	0.9600
N1—C8	1.366 (3)	C7—H7C	0.9600
N1—C9	1.392 (3)	C10—C11	1.380 (3)
N1—H1	0.8600	C10—C15	1.387 (3)
N2—C9	1.325 (3)	C11—C12	1.384 (3)
N2—C10	1.433 (3)	C11—H11	0.9300
N2—H2	0.8600	C12—C13	1.378 (3)
C1—C6	1.381 (3)	C12—H12	0.9300
C1—C2	1.381 (3)	C13—C14	1.381 (3)
C1—H1A	0.9300	C13—C16	1.511 (3)
C2—C3	1.367 (4)	C14—C15	1.383 (3)
C2—H2A	0.9300	C14—H14	0.9300
C3—C4	1.358 (4)	C15—C17	1.505 (3)
C3—H3	0.9300	C16—H16A	0.9600
C4—C5	1.386 (3)	C16—H16B	0.9600
C4—H4	0.9300	C16—H16C	0.9600
C5—C6	1.395 (3)	C17—H17A	0.9600
C5—C7	1.501 (3)	C17—H17B	0.9600
C6—C8	1.496 (3)	C17—H17C	0.9600
			
C8—N1—C9	129.81 (17)	N2—C9—N1	116.13 (18)
C8—N1—H1	115.1	N2—C9—S1	125.20 (16)
C9—N1—H1	115.1	N1—C9—S1	118.67 (15)
C9—N2—C10	124.43 (18)	C11—C10—C15	120.8 (2)
C9—N2—H2	117.8	C11—C10—N2	117.8 (2)
C10—N2—H2	117.8	C15—C10—N2	121.3 (2)
C6—C1—C2	120.2 (2)	C10—C11—C12	120.0 (2)
C6—C1—H1A	119.9	C10—C11—H11	120.0
C2—C1—H1A	119.9	C12—C11—H11	120.0
C3—C2—C1	119.4 (2)	C13—C12—C11	121.0 (2)
C3—C2—H2A	120.3	C13—C12—H12	119.5
C1—C2—H2A	120.3	C11—C12—H12	119.5
C4—C3—C2	120.7 (2)	C12—C13—C14	117.4 (2)
C4—C3—H3	119.7	C12—C13—C16	121.2 (2)
C2—C3—H3	119.7	C14—C13—C16	121.4 (2)
C3—C4—C5	121.6 (2)	C13—C14—C15	123.6 (2)
C3—C4—H4	119.2	C13—C14—H14	118.2
C5—C4—H4	119.2	C15—C14—H14	118.2
C4—C5—C6	117.7 (2)	C14—C15—C10	117.2 (2)
C4—C5—C7	119.4 (2)	C14—C15—C17	120.6 (2)
C6—C5—C7	122.9 (2)	C10—C15—C17	122.2 (2)
C1—C6—C5	120.4 (2)	C13—C16—H16A	109.5
C1—C6—C8	118.0 (2)	C13—C16—H16B	109.5
C5—C6—C8	121.57 (19)	H16A—C16—H16B	109.5
C5—C7—H7A	109.5	C13—C16—H16C	109.5
C5—C7—H7B	109.5	H16A—C16—H16C	109.5
H7A—C7—H7B	109.5	H16B—C16—H16C	109.5
C5—C7—H7C	109.5	C15—C17—H17A	109.5
H7A—C7—H7C	109.5	C15—C17—H17B	109.5
H7B—C7—H7C	109.5	H17A—C17—H17B	109.5
O1—C8—N1	123.27 (19)	C15—C17—H17C	109.5
O1—C8—C6	123.18 (19)	H17A—C17—H17C	109.5
N1—C8—C6	113.52 (18)	H17B—C17—H17C	109.5
			
C6—C1—C2—C3	−1.5 (4)	C10—N2—C9—S1	4.5 (3)
C1—C2—C3—C4	−0.3 (4)	C8—N1—C9—N2	5.5 (3)
C2—C3—C4—C5	1.2 (4)	C8—N1—C9—S1	−173.80 (17)
C3—C4—C5—C6	−0.2 (3)	C9—N2—C10—C11	108.4 (2)
C3—C4—C5—C7	−178.7 (3)	C9—N2—C10—C15	−74.2 (3)
C2—C1—C6—C5	2.5 (3)	C15—C10—C11—C12	1.9 (3)
C2—C1—C6—C8	−176.4 (2)	N2—C10—C11—C12	179.4 (2)
C4—C5—C6—C1	−1.7 (3)	C10—C11—C12—C13	−0.3 (4)
C7—C5—C6—C1	176.8 (2)	C11—C12—C13—C14	−1.3 (4)
C4—C5—C6—C8	177.2 (2)	C11—C12—C13—C16	179.6 (2)
C7—C5—C6—C8	−4.3 (3)	C12—C13—C14—C15	1.3 (4)
C9—N1—C8—O1	−8.8 (4)	C16—C13—C14—C15	−179.6 (2)
C9—N1—C8—C6	173.14 (19)	C13—C14—C15—C10	0.3 (3)
C1—C6—C8—O1	−57.8 (3)	C13—C14—C15—C17	−177.2 (2)
C5—C6—C8—O1	123.3 (3)	C11—C10—C15—C14	−1.9 (3)
C1—C6—C8—N1	120.3 (2)	N2—C10—C15—C14	−179.21 (19)
C5—C6—C8—N1	−58.6 (3)	C11—C10—C15—C17	175.5 (2)
C10—N2—C9—N1	−174.81 (18)	N2—C10—C15—C17	−1.8 (3)

### Hydrogen-bond geometry (Å, °)

**Table:**

*D*—H···*A*	*D*—H	H···*A*	*D*···*A*	*D*—H···*A*
N2—H2···O1	0.86	2.03	2.706	135
C17—H17B···S1	0.96	2.80	3.496	130
N1—H1···S1^i^	0.86	2.57	3.372	155

Symmetry codes:  (i) −*x*, −*y*+1, −*z*+1.
